# Transactivation of the estrogen receptor promoter by BRCA1

**DOI:** 10.1186/s12935-017-0401-2

**Published:** 2017-03-02

**Authors:** William B. Archey, Bradley A. Arrick

**Affiliations:** 1 Norris Cotton Cancer Center, 1 Medical Center Drive, Lebanon, NH 03755 USA; 2 Department of Medicine, Dartmouth Medical School, Hanover, NH 03755 USA

**Keywords:** BRCA1, Estrogen receptor, Human mammary cell lines, Transcriptional activation

## Abstract

**Background:**

Absence of the estrogen receptor-α (ER) is perhaps the most distinctive pathological feature of breast cancers arising in women who inherit a mutation in BRCA1. Two hypotheses, not necessarily mutually exclusive, exist in the literature that describe mechanisms of ER transcriptional repression in breast cancer. One hypothesis suggests that methylation of cytosine–guanine dinucleotides (CpGs) primarily mediates repression, while the other maintains that transcriptional control is mediated by certain positive and negative promoter elements.

**Methods:**

To determine if wild type BRCA1 could induce activity of the ER promoter, we performed a series of transient transfections with ER promoter segments linked to a luciferase reporter. The effect of BRCA1 on endogenous ER expression was evaluated by RNA analysis.

**Results:**

Following cotransfection with a BRCA1 expression plasmid, we observed that ER promoter-driven luciferase activity was significantly increased in both MCF10A and IMEC cells (*p* < 0.005 and 0.0005 respectively, two-tailed *t* test). Specifically, the full length ER promoter construct showed approximately 5.6-fold (MCF10A) and tenfold (IMEC) increases in luciferase activity following BRCA1 transfection, compared with transfection with an empty expression plasmid (i.e. lacking BRCA1 sequence). We localized the ER promoter segment responsible for transactivation by BRCA1 to a 109 bp region containing an AP2γ homologous site.

**Conclusions:**

The work described here, along with previously published work, indicates that activity of certain transcriptional regulatory elements and CpG methylation both represent important mechanisms by which the ER gene is typically inactive in breast cancers associated with BRCA1 mutations. The absence of ER in these breast cancers has significant implications for pathogenesis, prevention, and treatment.

## Background

One of the most distinctive biological features of breast cancers that arise in women who inherit an altered copy of the BRCA1 gene is a lack of expression of the ER [1–3]. The correlation between loss of BRCA1 and the ER-negative phenotype extends to sporadic (i.e. non-inherited) breast cancers as well [4–6]. Considering the importance of estrogen-based signaling in the genesis and progression of breast cancer, this characteristic of BRCA1-linked breast cancers has important consequences for the treatment and prevention of these cancers. We have therefore endeavored to elucidate the molecular basis of the ER-negative phenotype in breast cancers lacking BRCA1. Two non mutually exclusive mechanisms have been proposed to account for the lack of ER expression in some sporadic breast cancers, one epigenetic and the other transcriptional. Epigenetic silencing of ER expression by cytosine methylation within the CpG island associated with the 5′ region of the gene has been well documented in both cell lines and tumor tissue [7–9]. We have reported that CpG methylation of the ER gene is more extensive in ER-negative BRCA1-linked breast cancers compared with ER-negative breast cancers not linked to germline mutations in BRCA1 [10].

**Fig. 1:**
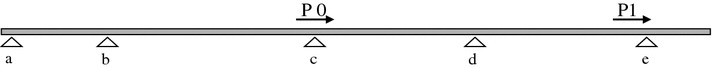
Topology of the ER promoter. The relative positions of the transcription initiation sites P1 and P0, as well as the documented regulatory elements are illustrated. The regulatory sites are indicated as follows: (*a*) ER-EH0 [18], (*b*) a negative regulatory element [17], (*c*) ERBF-1 [16], (*d*) ERUBF-1 [15], and (*e*) ERF-1 [13]

Expression of the ER can also be regulated at the transcriptional level, by the availability and activity of DNA-binding proteins that interact with portions of the ER promoter in a sequence-specific manner. The upstream promoter region of the ER has been reported to mediate transcription initiation from two alternative promoters, a proximal promoter termed P1 and a distal promoter called P0, yielding similarly sized mRNA transcripts with slightly different 5′ untranslated regions but identical coding regions. Analysis of ER-positive breast cancer cell lines and primary tumors indicates that the P1 promoter is the predominant site for initiation of transcription, but that the upstream P0 promoter may be active as well in some settings [11, 12]. Five groups have characterized distinct functional promoter elements, extending to ~4 kb upstream of P1. The most downstream of the elements, located within the 5′ noncoding region just upstream of the ATG start codon, is a positive element termed ERF-1 that binds the transcription factor AP2γ [13, 14]. Midway between P1 and P0 is another positive regulatory element, termed ERUBF-1, that functions as a transcriptional enhancer element in MCF-7 cells [15]. Hayashi et al. [12] have provided the clearest evidence that the upstream P0 promoter is also utilized in ER-positive breast cancers. They have described a transcriptional enhancer element in close proximity to this promoter that significantly augments transcription from P0-based promoter constructs in MCF-7 cells [16]. Furthermore, ER-positive cell lines that utilize P0 were shown to contain nuclear factors that specifically bind to this sequence, termed ERBF-1. A negative *cis* element, located ~3.2 kb upstream of P1, was identified via a strategy of transfecting cells with decoy fragments of dsDNA corresponding to segments of promoter sequence. Using this approach, Penolazzi et al. [17] reported that introduction of multiple copies of decoy dsDNA corresponding to a putative 102 bp negative element into MCF-7 or MDA-231 cells increased or reactivated ER expression, respectively. By RT-PCR, they showed that ER mRNA transcripts corresponding to the P0 promoter were the predominantly induced species, with undetectable levels of P1-derived ER mRNA. Finally, the most upstream positive enhancer element, termed ER-EH0 was mapped to a 35 bp segment beginning at −3744, relative to P1. Multiple DNA–protein complexes were demonstrated with this sequence, one of which included AP-1 [18]. The positions of these five regulatory elements, relative to the transcription start sites, are illustrated in Fig. 1.

We investigated transactivation of the ER promoter region by BRCA1 in two nontumorigenic mammary epithelial cell lines, MCF10A and IMEC. A series of ER promoter constructs were prepared and cotransfected with a BRCA1 expression plasmid. Here, we report that BRCA1 is able to transactivate the ER promoter in both MCF10A and IMEC cells and that a region of the ER promoter corresponding to a 109 bp segment located just upstream of the ERF-1 site is required for transcriptional activation by BRCA1. This region contains two binding sites for members of the AP2 family of transcription factors. Whether BRCA1′s effect is mediated through these elements, or via a putative BRCA1 response element, is the subject of on-going investigation.

## Methods

### Cell culture

MCF10A cells were obtained from the American Type Culture Collection (Manassas, VA). IMEC cells were kindly provided by Dr. James DiRenzo in the Department of Pharmacology and Toxicology at Dartmouth Medical School. Cells were cultured in DMEM:Ham’s F-12 medium, supplemented with 100 IU/ml of penicillin, 125 μg/ml of streptomycin, 2 mM l-glutamine, 10% fetal bovine serum, 20 ng/ml of epidermal growth factor, 0.5 μg/ml of hydrocortisone and 8 μg/ml of insulin.

### ER promoter constructs

ER promoter constructs were developed to facilitate localization of transcriptional control elements. They were generated from the previously published ER^**−**^3500-210Luc expression plasmid kindly provided by Weigel [13] (Department of Surgery, Stanford University). This construct includes a luciferase reporter (pGL2 Basic, Promega, Madison, WI) driven by ER promoter sequence from −3794 to +210 nucleotides relative to the first nucleotide of P1 [13]. In order to include the ER-EHO element documented by Tang et al. [18], we incorporated an additional 313 bp upstream fragment. Thus, the full length ER promoter construct, ER^**−**^3813-210Luc, now included 4023 bp of promoter sequence, properly positioned within the pGL2 Basic luciferase reporter.

5′ Deletion constructs of ER^**−**^3813-210Luc were made using two approaches. The first approach used combination endonuclease digestions with *Nhe*I, located in the upstream subcloning site of pGL2 Basic, along with either *Avr*II, *Pst*I, *Eco*RI, or *Nde*I. Plasmid fragment ends were blunted with Klenow fragment, and reactions were diluted out tenfold in order to favor intramolecular reactions before religation using T4 DNA ligase. The 5′ deletion constructs created from ER^**−**^3813-210Luc in this manner were as follows: ER^**−**^2641-210Luc (*Avr*II), ER^**−**^1231-210Luc (*Pst*I), ER^**−**^743-210Luc (*Eco*RI) and ER^**−**^42-210Luc (*Nde*I).

Because we could not find additional unique restriction sites within ER^**−**^42-210Luc, we used a second approach to produce further promoter deletion constructs. Unidirectional deletions of ER^**−**^42-210Luc were made by taking advantage of the unique property of *Exo*III nuclease to efficiently digest 5′ overhangs, while leaving 3′ overhangs undigested. In brief, 2.5 μg of *Kpn*I-*Mlu*I digested ER^**−**^42-210Luc DNA was subjected to digestion with 150 U of *Exo*III at 15 °C. Aliquots of 3 μl (~300 ng DNA) were removed from the *Exo*III digestion reaction at four time intervals (1, 20, 40, and 60 s), and placed directly into 16.5 μl of H_2_O preheated to 75 °C. Permanent inactivation of *Exo*III was effected by a 20 min incubation at 75 °C. Cleavage of the 5′ overhangs created by *Exo*III digestion was effected by the addition of 4 U of S_1_ nuclease and incubation at room temperature for 20 min. The S_1_ nuclease reaction was halted by the addition of 5 μl of STOP buffer (800 mM Tris, 20 mM EDTA, 80 mM MgCl_2_, pH 8.0). Two units of Klenow fragment and 10 μM dNTPs were then added to blunt the 3′-overhangs, followed by plasmid ligation with T4 DNA ligase. Plasmid DNA from transformed bacteria (DH5α cells) was sequenced to confirm isolation of the deletion constructs of interest. The following promoter deletion constructs were generated using this method: ER^**−**^24-210Luc, ER14-210Luc, ER62-210Luc, and ER171-210Luc. All promoter constructs were sequenced.

### Transfections and luciferase assays

Luciferase experiments involved cotransfection of cells with either a BRCA1 expression plasmid or empty vector (pRK7, Genentech, S. San Francisco, CA), along with one of the ER promoter constructs. The BRCA1 expression plasmid was prepared by subcloning the full length BRCA1 cDNA into a pRK7 backbone previously described [19]. Cotransfection of either BRCA1 or pRK7, along with the empty pGL2 Basic vector was also included in each experiment. Three microliters of FuGENE 6 Transfection Reagent (Roche, Indianapolis, IN) was combined with 1.5 μg total DNA for incubation with adherent cells in a 9.6 cm^2^ plate. The ratio of luciferase reporter to expression plasmid DNA was 1:4. Cells were 45–55% confluent at transfection and 80–90% confluent at time of harvest. Cell media was changed at 24 h, and cells were harvested after 48 h.

Transfected cells were harvested on ice with lysis buffer (25 mM glycylglycine, 4 mM EGTA, 15 mM MgSO_4_, 1% Triton-X, and 0.1 mM DTT, pH 7.8), which was added directly to the cell culture dish following media aspiration and 2 washes with cold phosphate-buffered saline (PBS). Dishes were subsequently scraped and lysates transferred to pre-chilled tubes. Cell lysates were centrifuged at 9000*g* and supernatants were stored at −80 °C until analysis. In a 96-well plate, 40 μl of each lysate was analyzed for luciferase activity in the presence of 145 μl of assay buffer (25 mM glycylglycine, 15 mM KPO_4_, 15 mM MgSO_4_, 4 mM EGTA, 25 mM ATP, and 1 mM DTT, pH 7.8), following addition of 40 μl luciferin reagent (400 μM luciferin, 25 mM glycylglycine). Each lysate was also evaluated for protein levels, in triplicate, using the BCA Protein Assay (Pierce, Rockford, IL). Luciferase/protein for cells transfected with each ER promoter construct, and either BRCA1 or pRK7, were normalized with lysate from cells transfected with the empty pGL2 Basic vector.

### RT-PCR

Cell line RNA was isolated by first treating adherent cells with 5.7 ml of TRIzol Reagent (Invitrogen, Carlsbad, CA), and then scraping the solublized cell debris from a 10 cm^2^ dish. TRIzol-treated cell solution was incubated for 15 min at room temperature, and then treated with 1.4 ml of chloroform. After vigorous agitation, the chloroform-TRIzol solution was centrifuged and the aqueous phase transferred to a tube containing 2.85 ml of isopropanol. The sample was then mixed and incubated at room temperature for 10 min. Following centrifugation the RNA pellet was washed with 70% ethanol and dried under vacuum. The RNA pellet was then reconstituted in H_2_O previously treated with diethyl pyrocarbonate, a potent inactivator of RNase.

Using 2 μg of RNA as template, each reverse transcriptase (RT) reaction included 1× AMV buffer (Roche, Indianapolis, IN), 50 μg/ml BSA (NE Biolabs, Beverly, MA), 0.4 μM random hexamers (Perkin Elmer Corp., Foster City, CA), 200 μM each dNTP, 0.39 U/μl RNase inhibitor (Promega, Madison, WI), and 400 U MMLV RT (Gibco, Gaithersburg, MD). Reactions were incubated at 37 °C for 2 h, followed by heat inactivation of the RT at 75 °C for 5 min. To confirm the presence of equal amounts of cDNA among the RNA samples, PCR amplification of human β-actin was used as an initial control reaction. The β-actin primers were as follows: 5′-GGGACCTGACCGACTACCTC-3′ and 5′-GGGCGATGATCTTGATCTTC-3′.

β-actin PCR reactions included 1/10 of the RT reaction as template, along with 1× PCR buffer (Gibco, Gaithersburg, MD), 2.5 mM MgCl_2_, 200 μM each dNTP, 125 ng each primer, and 1.5 U *Taq* Platinum. The reaction conditions consisted of initial denaturation at 95 °C for 5 min, followed by 18 cycles of 94 °C for 30 s, 52 °C for 30 s, and 72 °C for 30 s. The PCR profile ended with a final extension phase of 72 °C for 10 min. β-actin PCR products were then run out on a TBE-agarose gel to confirm approximately equal loading of RNA templates among sample reactions.

ER PCR was performed with the following primers: 5′-CTATATGTGTCCAGCCACCAACC-3′ and 5′-CTCTACACATTTTCCCTGGTTCCT-3′. The upper primer corresponded to sequence in exon 3 of ER, while the lower primer corresponded to sequence in exon 6 [17]. Therefore, these primers were specific for cDNA amplified from RNA during the RT reaction. Using 1/10 of the RT reaction as template, each ER PCR mixture included 1× PCR buffer, 1.5 mM MgCl_2_, 200 μM each dNTP, 0.5 μM each primer, and 1.5 U *Taq* Platinum. The reaction conditions consisted of initial denaturation at 95 °C for 5 min, followed by 38 cycles of 94 °C for 1 min, 57 °C for 1 min, and 72 °C for 1 min. The PCR profile ended with a final extension phase of 72 °C for 10 min. ER PCR products were then run out on a TBE-agarose gel to confirm the presence or absence of product.

### Statistical analysis

**Fig. 2:**
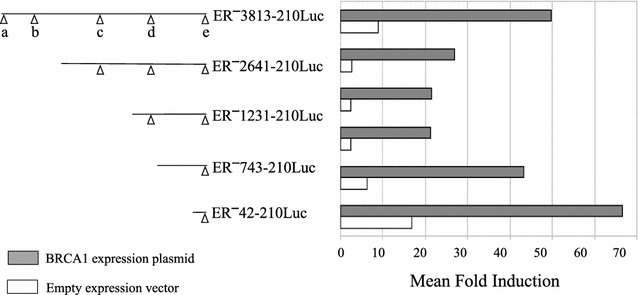
Transcriptional activation of the ER promoter by BRCA1. Promoter activity for a series of ER promoter constructs with progressive 5′ deletions designed to sequentially remove the established regulatory sites. Fold induction in luciferase activity (*x axis*) is shown for each of the ER promoter constructs following transfection with either a BRCA1 expression plasmid (*filled bars*) or the empty pRK7 vector (*open bars*). The fold induction in luciferase activity is the number of photon units per unit time of data capture (RLU) divided by protein, normalized by the corresponding value from cells transfected with the empty pGL2 Basic vector. RLU/protein values from the empty pGL2 Basic vector ranged from ~35 to ~400, while those from the ER promoter constructs were >1500. Data shown represents the average of up to 7 experiments, with luciferase and protein measurements performed in triplicate for each experiment

**Fig. 3:**
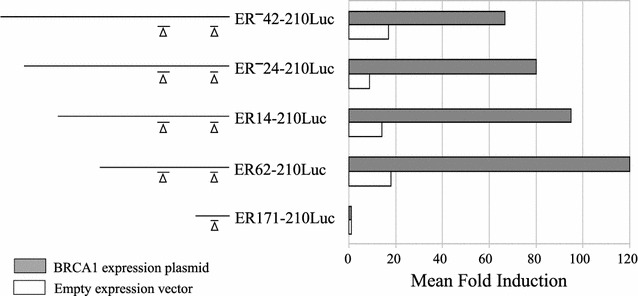
Transcriptional activation by BRCA1 with a series of *Exo*III-generated ER promoter luciferase constructs. Luciferase activity for *Exo*III-generated ER promoter constructs in MCF10A cells. Fold induction in luciferase activity (*x axis*) is shown for ER promoter constructs following co-transfection with either a BRCA1 expression plasmid (*filled bars*) or the empty pRK7 vector (*open bars*). Fold induction values were calculated as in Fig. 2. Data shown represents the average of 2–7 experiments, with luciferase and protein measurements performed in triplicate for each experiment

**Fig. 4:**
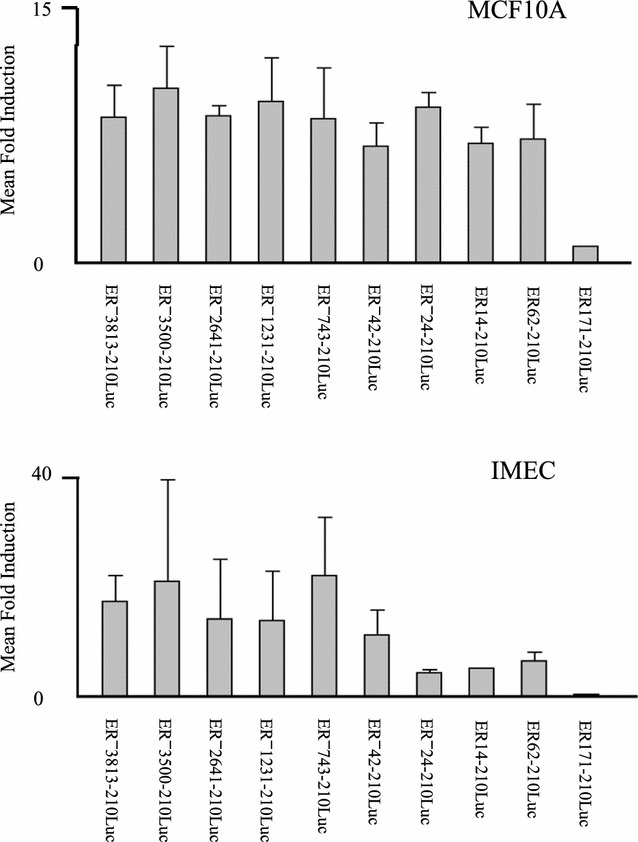
Localization of the segment of ER promoter mediating transactivation by BRCA1. Mean fold induction in luciferase activity (*y axis*) by BRCA1 in MCF10A cells (*top panel*) and IMEC cells (*bottom panel*) is indicated for all ER promoter constructs (*x axis*), calculated as the ratio of promoter activity when co-transfected with BRCA1, divided by promoter activity measured with co-transfection of pRK7. Standard error of the mean is indicated

Significance of luciferase activity among BRCA1- and pRK7-transfected cells in Figs. 2, 3, and 4 was calculated using a two-tailed student *t* test. Normalized luciferase activity in BRCA1 transfections and pRK7 transfections were paired for each experiment. Among the ER luciferase constructs, the mean induction by BRCA1 on luciferase activity was analyzed for significance using an unpaired ANOVA analysis.

## Results

To focus our examination of transactivation of the ER promoter by BRCA1 in a cell type relevant to its role as a tumor suppressor gene in breast cancer, we performed all experiments with two human nontumorigenic mammary epithelial cell lines. The MCF10A cell line was used for the majority of the experiments because of its ease of transfection (consistently >50%), low endogenous level of wild type BRCA1 expression, and a wild type *p53* status [20, 21]. In parallel, transactivation of the ER promoter was examined using an immortalized nontumorigenic mammary epithelial cell line with wild type BRCA1, IMEC. IMEC cells were generated by introduction of a recombinant retrovirus expressing the catalytic subunit of human telomerase into primary human mammary epithelial cells [22]. Our initial experiments tested the hypothesis that BRCA1 could transactivate the ER promoter. To test this hypothesis, we extended a previously described ER promoter-luciferase construct, ER^**−**^3500-210Luc [13], to include 3813 nucleotides upstream of P1. As shown in Fig. 1, this construct included all five of the documented transcriptional regulatory elements in the ER promoter region. This full length construct, labeled ER^**−**^3813-210Luc, was cotransfected along with a BRCA1 expression plasmid, or the empty expression vector pRK7. Figure 2 shows the luciferase activity for ER^**−**^3813-210Luc in MCF10A cells, demonstrating a significant augmentation (*p* < 0.005) in ER promoter transactivation when cotransfected with the BRCA1 expression plasmid. We observed a 50-fold increase over the empty luciferase vector when cotransfected with BRCA1, as compared with a ninefold increase when cotransfected with the empty expression plasmid, pRK7. It is possible that the observed luciferase activity from the ER promoter constructs when cotransfected with the pRK7 plasmid was due to the effect of endogenous BRCA1. IMEC cells also exhibited a significant transactivation of the full-length promoter construct, with a 29.4-fold and 3.3-fold increase when co-transfected with the BRCA1 and pRK7 expression plasmids, respectively (*p* < 0.0005).

Having observed transactivation of our full length ER promoter construct by BRCA1 in two ER-negative cell lines, we proceeded to test a series of deletion constructs designed to sequentially remove the five documented transcriptional regulatory sites shown in Fig. 1. These deletion constructs, depicted in the left-hand portion of Fig. 2, were cotransfected with either BRCA1 or pRK7, as before. The normalized luciferase values are shown in the right-hand portion of Fig. 2. Although there was some variation in the magnitude of induction of luciferase activity by BRCA1, when compared with the induction seen with the empty pRK7 plasmid there were no significant differences between the constructs (*p* > 0.9 for both MCF10A and IMEC cells). These data suggested that the segment of the ER promoter which mediates transactivation by BRCA1 was contained within the region extending from 42 bp upstream to 210 bp downstream of the P1 transcriptional start site.

In order to localize more precisely the segment of promoter sequence in proximity to P1 responsible for BRCA1 transactivation, we prepared additional deletion constructs by limited *Exo*III nuclease digestion of the ER^**−**^42-210Luc construct. These constructs and their promoter activity following cotransfection with either BRCA1 or pRK7 in MCF10A cells are shown in Fig. 3. Induction of promoter activity by BRCA1 was not significantly diminished by removal of up to 103 nucleotides from the 5′ end of the ER^**−**^42-210Luc construct (*p* > 0.8 for the series ER^**−**^42-210Luc, ER^**−**^24-210Luc, ER14-210Luc, and ER62-201Luc). However, as indicated in Fig. 3, the ER171-210Luc construct did not show any induction of luciferase activity upon transfection with either BRCA1 or pRK7. The fold inductions obtained with co-transfection of the BRCA1 expression plasmid for the entire ER promoter deletion series in both MCF10A and IMEC cells is illustrated in Fig. 4. These data clearly indicate that the specific region of the ER promoter mediating transactivation by BRCA1 is located between 62 and 171 bp downstream of the P1 transcriptional start site.

**Fig. 5:**
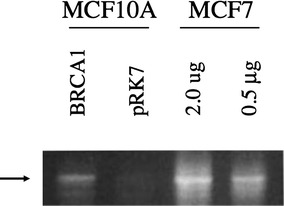
Expression of ER mRNA following transfection with BRCA1 or pRK7. Cells were harvested 48 h after transfection and mRNA for RT-PCR analysis were prepared. PCR products from the RT-PCR analysis were run on an agarose gel for analysis of ER mRNA, with the products appearing at the expected size of 603 bp (*arrow*). Reverse transcriptase (RT) reactions were performed with or without RT to control for contamination, and mRNA prepared from untransfected MCF7 cells serving as positive control

To determine if the induction of ER promoter activity by BRCA1 was seen at the level of the endogenous gene (i.e. at the mRNA level), we performed RT-PCR on extracts isolated following transfection with BRCA1 or pRK7. Figure 5 indicates that ER mRNA is barely detectable in pRK7-transfected mRNA, as would be expected in ER-negative cell lines. Following transfection with BRCA1, MCF10A cells showed an increased level of ER mRNA, suggesting that the endogenous ER promoter was transactivated. Preparation of mRNA from an untransfected ER-positive cell line, MCF7, was used as a control. Figure 5 illustrates that although BRCA1 induces the production of ER mRNA in MCF10A cells, the levels are still much lower than in an ER-positive cell line. Induction of ER mRNA by BRCA1 was not detectable in IMEC cells (not shown). This was contrary to expectation considering that our previous luciferase results showed BRCA1 could transactivate ER promoter activity in both MCF10A and IMEC cells, and may be a function of epigenetic silencing of the endogenous ER promoter in IMEC cells.

## Discussion

Investigators have reported that ER transcriptional regulation is at least in part influenced by the activity of certain *cis*- and *trans*-acting factors in breast cancer cell lines [13–18]. Inherited primary breast cancers that are characterized by a mutation in the tumor suppressor gene, BRCA1, do not express ER in two-thirds to 90% of patients [1–3]. Therefore, we wanted to determine if ectopic levels of BRCA1 could influence the activity of the ER promoter, potentially through the regulation of these transcriptional sites. The data indicated that in fact BRCA1 could transactivate an ER promoter-containing construct driving luciferase expression in two nontumorigenic, ER-negative, cell lines.

The 109 bp portion of P1 found to mediate BRCA1 transactivation of ER did not correspond to any of the documented transcriptional sites in the ER gene. This 109 bp segment was located just upstream of the ERF-1 site first described by DeConinck et al. [13], and included a nearby site homologous to ERF-1 previously shown to bind with less affinity to AP2γ. This homologous ERF-1 site is located between positions 130 and 149. Following a search of a transcription factor data base (Transfac: Transcription Factor Database, http://transfac.gbf.de/TRANSFAC/) with the sequence of the 109 bp P1 region transactivated by BRCA1, we noted an additional AP2 site present at position 64–75. Not identified were any of the DNA-binding sequences for BRCA1 reported by Cable et al. [23], or an Oct-1 binding site implicated by Hosey et al. [24] as important to BRCA1’s impact on ER expression.

Induction of the ER endogenous gene by BRCA1 was evident in MCF10A cells, as shown in Fig. 5. This observation is consistent with the report of Hosey et al. [24] that expression of wild type BRCA1 in a BRCA1-mutated and ER-negative breast cancer cell line (HCC1937) resulted in detectable ER mRNA levels. Of note, transient transfection of BRCA1 in MCF10A cells was not sufficient to induce ER mRNA to the level of an ER-positive cell line.

## Conclusion

The role of BRCA1 in the transcriptional regulation of ER is a critical question in breast cancer research, with implications for both prevention and treatment. The work presented here demonstrates the importance of a CpG-rich 109 bp segment in the transactivation of the ER promoter by BRCA1 in two transformed non-tumorigenic human mammary epithelial cell lines. Our observations with IMEC cells suggest that methylation within this region may constitute an important mechanism of epigenetic control that affects the ability of BRCA1 to induce the endogeneous ER gene’s promoter activity. If true, we would predict that the addition of a demethylating agent such as azacytidine would allow for detectable activation of the endogenous gene by BRCA1. A notable limitation to this work is that transformed cell lines with wild type BRCA1 were studied, and not primary cells or BRCA1-null cell lines. Use of cells completely lacking endogenous BRCA1 would facilitate experiments correlating this effect of BRCA1 with its disease-causing mutations, thus linking our observations with critical aspects of pathogenesis of breast cancer in BRCA1 mutation carriers.

## Abbreviations

ER: estrogen receptor-α
CpGs: cytosine–guanine dinucleotides
RT: reverse transcriptase
